# Comments on Two Recent Cases of Gastro-Enterostomy

**Published:** 1909-02

**Authors:** Edward G. Jones

**Affiliations:** Atlanta, Ga.; Professor of Surgery Atlanta School of Medicine


### COMMENTS ON TWO RECENT CASES OF GASTRO-ENTEROSTOMY.*

BY EDWARD G. JONES, M. D., PROFESSOR OF SURGERY ATLANTA SCHOOL OF MEDICINE.

While the cases I shall report suggest a discussion of many of those interesting questions connected with gastric surgery, they are presented as illustrative of two points particularly:

(1) The usual curability of chronic gastric and duodenal ulcers by surgery as compared with their usual incurability by medical means, and

(2) Our present inability to predicate the exact character of the lesion in certain cases of gastric hemorrhage.

It may be recalled that, in reporting some cases of gastro-enterostomy, I presented before this Society last May, a man operated on for chronic gastric ulcer a year ago. He remains entirely well, has no uncomfortable symptoms whatever, and has gained 50 pounds.

1.—Chronic Duodenal Ulcer. Male, age 29. Family history unimportant. Indigestion for 15 years. Five or six years ago attacks were periodic, lasting from one to three weeks; now patient always suffers at least epigastric discomfort if enough is eaten to satisfy appetite. On this account he constantly underfeeds. Six weeks before coming to me he had Hematemesis; one year before he also vomited blood. Following both the attacks of hematemesis and also on perhaps a half dozen other occasions during the past three years, the attacks of Melena were observed. Patient complained of Hyperacidity, takes soda and avoids pickles, lemons, tomatoes, etc., because he has learned that

*Presented before the Fulton County Medical Society, December 3, 1908.

such things induce a "boiling" sensation in his stomach. He frequently wakes after midnight or early in the morning feeling that he must regurgitate a sour collection in his stomach. If he is able to do this he feels relieved; if not, his distress continues. Because he had had a comparatively recent attack of hematemesis and had never taken the tube it was thought wise not to confirm these presumptive evidences of hyperacidity. His teeth have been eroded and "worn to the quick," probably by acid eructations. The distress is more of a epigastric tenderness than actual pain. It is relieved to a great extent by vomiting, though patient has learned that the necessity for vomiting may be avoided to a great extent by taking small amounts of food. Flatulence is not constant, but common. The patient is thin and anemic, and his general discomfort is such as to incapacitate him for work. Examination by cautious dilatation of the stomach (?) revealed no evidence of pyloric stricture.

A diagnosis of gastric or duodenal ulcer was made. The sex, the frequent attacks of melena as compared with hematemesis, the incidence of distress after midnight, the prior periodicity of the attacks suggested duodenal location.

Operation revealed an ulcer scar, indurated and whitish, just beyond the pyloric vein, and having a base comprising about a square inch. The scar being anterior, Finney's duodenostomy was decided against and posterior gastro-jejunestomy was done. The meso-colic band was long and pulled the beginning jejunum toward the median line. It was snipped with scissors and the anastomosis established in the oblique line, commonly followed by the Mayos. Moynihan and Mayo Robson, under these circumstances, make the stomach incision vertical, believing that when the jejunus thus takes a course directly downward, there is less danger of troublesome kinking of the gut.

The patient recovered promptly, and has been entirely relieved of his previous discomfort. A report two months after operation says that he feels better than he has in years, and has gained 29 pounds in weight. He has not vomited since the operation—not even from the ether.

Given a diagnosis of gastric ulcer supported by a first attack of hematemesis, will the patient usually be cured by medical treatment? And by the term "cured" is not meant merely the stopping of the hemorrhage; as a matter of clinical history, we know that the hemorrhage will commonly stop at least temporarily. But does the patient, or does he not, continue to suffer with intermittent or constant dyspepsia, epigastric discomfort, "heartburn," flatulence, etc., and does the hemorrhage, if there have been hemorrhage, recur after weeks or months?

That in the majority of instances such patients will not be cured by medical treatment alone; a multitude of authorities, both medical and surgical, might be called to witness. Bulstrode, in 1902, analyzing 500 cases, admitted to the London hospital, concluded that at least two-fifths of these patients (who had been treated medically) were suffering from recurrences or relapses—and these figures did not include patients who came in suffering from the complications and sequelae of ulcer. Paterson traced a series of 72 cases treated medically, and discharged as cured. He believes that at the time of observation all but 10 had (a) suffered from one to four recurrences of hematemesis, or (b) died of hematemesis or of some other accident traceable to the stomach lesion. Moynihan maintains that the percentage of permanent cures of gastric ulcer medically treated is under 25. Mayo Robson believes that at least half of all patients suffering from stomach ulcer and treated medically, ultimately succumb to the disease or one of its complications—without reference to the considerable number of persons so affected who lead more or less miserable lives and fall victims to other maladies because of their lowered vitality.

Does latter day surgical treatment of these lesions offer any more encouragement than is contained in the above reports? In answer to that question I submit the following:

Mayo Robson, reporting upward of 300 cases treated by gastro-enterostomy, has had a mortality of a little above one per cent.; and more than 90 per cent. of his patients so treated remained completely relieved of all symptoms up to the time of his report, which covered about five years.

The Mayos report this year "of the 318 actually demonstrated ulcers, we have traced 234. 80.7 per cent. are cured; 9 per cent. are improved; 4.2 per cent. are unimproved; 6 per cent. have died since operation for various causes (but in only two of these deaths was the stomach concerned.") A total of 89.7 per cent. were, therefore, cured and improved.

While the actual statistics of Moynihan are not at hand, it is known that his experience is practically the same as that set forth above.

It is especially to be remembered that these statistics include the early as well as the recent cases of these surgeons; it is the early cases which furnish the failures to a very large extent because these men had to work out the proper technic of the operation themselves. Certain it is that any criticism based upon the inclusion in their statistics of cases operated on too recently to be yet classed as cured is more than offset by the experimental nature of their early experience.

We may, therefore, conclude upon ample evidence that surgery which a few years ago was invoked only to treat the sequeble of stomach ulcer, (stricture, perforation, hemorrhage, etc.,) may now properly be invoked to cure the indigestion traceable to the ulcer.

2.—Multiple Acute Ulcers. Female, age 53. On November 19, 1908, suffered with a severe attack of hematemesis. She was seen by Dr. Dorsey soon afterward. She rallied well under appropriate medical treatment. On November 21, she suffered another attack of hematemesis, vomiting perhaps, three pints of blood. I saw her two hours later, with Dr. Dorsey. She was blanched and in collapse, this state of collapse having come on with the hematemesis—not gradually before vomiting began.

It was not through that the patient had a ruptured aneurysm; she was not arterio-sclerotic; there was no enlargement of the liver or spleen; there was no history of hemophilia or vicarious menstruation; nor was there any previous history characteristic of chronic ulcer except a possible hyperacidity and an indifinite legend of melena just a year before. The rally after the first hemorrhage, the somewhat sudden appearance of collapse with the second hemorrhage, the fact that perhaps the major portion of blood vomited on both occasions was not clotted, but liquid, and the history of possible melena led us rather to believe that there had been erosion of a vessel of some size in connection with an old ulcer instead of capillary oozing.

It was decided to wait in the hope that the patient would rally. Twelve hours later, however, when there was little evidence of improvement in the woman's condition, it was decided to operate in the hope that a controllable lesion might be discovered and properly handled.

The abdomen having been opened, the stomach was hastily examined externally for an ulcer site which might be excised or otherwise properly treated. No lesion being apparent, the stomach was opened. A quart of blood was quickly removed, mostly clotted. Upon close examination of the mucous membrane, numerous small points having a diameter of one-fourth inch and downward were discovered from which blood slowly oozed. Unfortunately the welfare of the patient prohibited a close study of any of these areas, but the impression was the same as if the epidermis had been brushed off by some light blow and capillary bleeding had come on in a few seconds. No extensive lesion being found, it was apparent that the hemorrhage had its origin in these small areas. The condition of the patient being very precarious, no time was lost to discover how many such bleeding points could be found. Not less than a half dozen, mostly on the posterior surface but involving also the anterior wall, were easily seen. In addition there were seen numerous small hemorrhagic "punctate spots" where blood had apparently clotted in the small vessels, and where one would believe that hemorrhage had previously taken place, but had now stopped.

It being manifestly impossible to control the bleeding by direct treatment, the stomach was closed and a posterior gastro-jejunostomy quickly performed. The patient was returned to bed practically pulseless, though the operation has been short. Intravenous infusion of saline was begun concurrently with the laparotomy. Saline per rectum, together with the usual methods of stimulation, were continued after she was put to bed. The patient died, however, four hours afterward, not having vomited blood meantime.

While it is not intended to bring within the scope of this report the question as to when, if at all, it is advisable to operate for gastric hemorrhage, it is not out of place to say that in this instance the practice of the highest authorities was followed. It may also be said that no operative procedure except a gastro-enterostomy could have commended itself in this case; but I believe that an early gastro-enterostomy would stop hemorrhage from these lessions by emptying the stomach (thus relieving the stretched mucous membrane) and by setting it entirely at rest. Indeed clinically this procedure alone has proved so efficacious in stopping hemorrhage that Mayo Robson and Moynihan state that they seldom feel called upon to do anything else (surgically) to stop gastric hemorrhage. However, in waiting the advice of excision or direct ligation, or any other such measure is adopted they invariably supplement it with gastro-enterostomy to secure rest.

To me it appears that if in this instance we had operated immediately after the first hemorrhage we would probably have saved the woman's life—basing this belief on what is said by experienced clinicians about the competence of gastro-enterostomy to stop gastric hemorrhage. However, in waiting the advice of all authorities, medical and surgical, was complied with, and under the same circumstances with the same light I would wait again.

With reference to our foreknowledge of the character of the lesion in this case, it may be said that a large unheralded spontaneous gastric hemorrhage most usually betrays one or the other of the several lesions commonly catalogued as acute ulcer, but gives little information as to whether there is capillary oozing from one or more areas or bleeding from a comparatively large vessel. Mayo Robson says: "Capillary hemorrhage may be so free as to render it difficult to say that some large vessel has not given way, yet after death a careful examination may fail to discover any gross vascular lesion * * * Arterial bleeding is mostly responsible for the serious and fatal hematemesis from gastric ulcer." But the same author adds, "In the present state of our knowledge it is impossible to diagnose the size of the vessel perforated, either from the amount of blood or the length of survival * * * If, therefore, medical treatment and rest properly carried out are not successful in arresting the bleeding in a few hours, or if after being arrested it recurs, we should be driven to the conclusion that a large vessel is perforated."

205 Fourth National Bank Building.

DISCUSSION OF DR. JONES' PAPER.

Dr. Dorseey said that melena as a symptom was far more likely to indicate piles than gastric ulcer. Hematemesis was not clearly diagnostic, because in one of his cases the blood did not all sink in water; it seemed too fresh. Pulmonary signs were lacking and bile followed the bleeding, which showed the gastric source.

He described a case of hematemesis in pregnancy occurring at third or fourth month of pregnancy three times. She aborted twice and recovered perfect health twice. She is now pregnant and vomiting blood the third time. He asks when he shall operate on the stomach.

Dr. Barnett related the case of a man thirty years old who passed large amounts of blood by the mouth and rectum. He had no pulmonary trouble and no blood disease. He had had two attacks of malaria obscure in history. Seemed in good health. Had three or four hemorrhages in past three years increasing in frequency. He died without operation. No post-mortem could be obtained.

Dr. Strickler said there was no evidence of leukaemia in the previous case. He had examined the blood personally.

He believes that all these cases should be treated surgically if medicine doesn't give immediate relief.

He was most unhappily impressed by a case of classic doudenal ulcer. The man improved under treatment and was apparently doing nicely for several days. He called on the doctor hurriedly one day and collapsed. He died in an hour from shock.

Dr. Duncan said that soda was greatly abused and even dangerous household remedy, because it was so often taken to neutralize hyperacidity when it really increased this condition.

Dr. Clark asked Dr. Barnett as to the temperature in his case. Dr. Barnett said it was slightly subnormal most of the time, though once it ran to 100.

Dr. Cartlege asked Dr. Jones if one of the cases he described in which a mother had been nursing a tuberculous son could be pulmonary in origin.

Dr. Jones replying, said there was no exidence of pulmonary difficulty.

Speaking of the technique of the operation, he said: the jejunum may go to the right or to the left or directly downward and the line of the posterior incision in the stomach should correspond to this varying direction however it might be in each individual case.

The majority of ulcers are really in the duodenum. The pouching of tissues makes it appear in the stomach, when really it is further down.

